# Supplementary material for: Metabolic and bariatric surgery among patients with social anxiety disorder, a matched cohort study
Source: PLoS One. 2026 Jan 27;21(1):e0341175. doi: 10.1371/journal.pone.0341175 (PMC12843590; doi:10.1371/journal.pone.0341175)
Supplement: S1 File — Table S1. Baseline characteristics of patients with social anxiety disorder and matched controls undergoing metabolic and bariatric surgery. Table S2. Postoperative complications within 30 days after surgery among patients with social anxiety disorder and a matched control group. Table S3. Baseline characteristics of patients with social anxiety disorder and matched controls undergoing metabolic and bariatric surgery, stratified by surgical method. Table S4. Weight loss at 1 and 2 years after Roux-en-Y gastric bypass surgery among patients with social anxiety disorder and a matched control group. Table S5. Weight loss at 1 and 2 years after sleeve gastrectomy among patients with social anxiety disorder and a matched control group. Table S6. Risk for alcohol or substance abuse, and self-harm events after surgery among patients with social anxiety disorder compared to a matched control group, stratified by surgical method. Table S7. Health-related quality of life estimated with the Obesity related problem scale before and after Roux-en-Y gastric bypass surgery among patients with social anxiety disorder and a matched control group. Table S8. Health-related quality of life estimated with the Obesity related problem scale before and after Sleeve gastrectomy among patients with social anxiety disorder and a matched control group. (DOCX) [file pone.0341175.s001.docx]

| **Table S1**. Baseline characteristics of patients with social anxiety disorder and matched controls undergoing bariatric surgery | | | |
| --- | --- | --- | --- |
|  | Social anxiety group | Control group | *Standardized mean difference* |
| *N* | *586* | *59528* |  |
| *Age, years* | *36.4 ± 10.2* | *41.0 ± 11.1* | *0.431* |
| *Body Mass Index, kg/m^2^* | *43.2 ± 6.2* | *42.0 ± 5.5* | *0.206* |
| ***Sex*** |  |  |  |
| *Men* | *114 (19.5%)* | *14102 (23.7%)* | *0.102* |
| *Women* | *472 (80.5%)* | *45426 (76.3%)* | *0.102* |
| ***Comorbidities*** |  |  |  |
| *Type-2 diabetes* | *80 (13.7%)* | *8123 (13.6%)* | *0.003* |
| *Hypertension* | *93 (15.9%)* | *14833 (24.9%)* | *0.225* |
| *Sleep apnea* | *51 (8.7%)* | *5711 (9.6%)* | *0.031* |
| *Dyslipidemia* | *48 (8.2%)* | *5687 (9.6%)* | *0.049* |
| *Chronic Obstructive Pulmonary Disorder* | *48 (3.8%)* | *1812 (3.0%)* | *0.044* |
| *Cardiovascular disease* | *10 (1.7%)* | *2675 (4.5%)* | *0.162* |
| ***Socioeconomic factors*** |  |  |  |
| *Disposable income* |  |  |  |
| *Quartile 1* | *330 (56.3%)* | *14938 (25.1%)* | *0.670* |
| *Quartile 2* | *167 (28.5%)* | *15051 (25.3%)* | *0.072* |
| *Quartile 3* | *60 (10.2%)* | *14889 (25.0%)* | *0.396* |
| *Quartile 4* | *29 (4.9%)* | *14647 (24.6%)* | *0.578* |
| *Unemployment* | *67 (11.4%)* | *6179 (10.4%)* | *0.032* |
| ***Origin*** |  |  |  |
| *Swedish born, Swedish-born parents* | *524 (89.4%)* | *47211 (79.3%)* | *0.281* |
| *2^st^ generation immigrant* | *23 (3.9%)* | *3031 (5.1%)* | *0.058* |
| *1^st^ generation immigrant* | *39 (6.7%)* | *9283 (15.6%)* | *0.286* |
| ***Surgical method*** |  |  |  |
| *Roux-en-Y gastric bypass* | *501 (85.5%)* | *51854 (87.1%)* | *0.047* |
| *Sleeve gastrectomy* | *85 (14.5%)* | *7674 (12.9%)* | *0.047* |
| ***Surgical access*** |  |  |  |
| *Laparoscopy* | *565 (96.4%)* | *58017 (97.5%)* | *0.064* |
| *Conversion to open surgery* | *4 (0.7%)* | *349 (0.6%)* | *0.012* |
| *Primary open surgery* | *17 (2.9%)* | *1162 (2.0%)* | *0.058* |

| **Table S2.** Postoperative complications within 30 days after surgery among patients with social anxiety disorder and a matched control group. | | | |
| --- | --- | --- | --- |
|  | Social anxiety group | Control group | P* |
| Postoperative complications | 69 (12.1%) | 451 (8.0%) | <0.001 |
| *Bleeding* | *13 (2.3%)* | *103 (1.8%)* | *0.436* |
| *Leak/intraabdominal abscess* | *7 (1.2%)* | *79 (1.4%)* | *0.768* |
| *Wound complication* | *16 (2.8%)* | *78 (1.4%)* | *0.006* |
| *Bowel obstruction or ileus* | *7 (1.2%)* | *71 (1.3%)* | *0.974* |
| *Marginal ulcer* | *4 (0.7%)* | *21 (0.4%)* | *0.273* |
| *Pulmonary complication* | *4 (0.7%)* | *25 (0.4%)* | *0.386* |
| *Cardiovascular complication* | *0 (0.0%)* | *6 (0.1%)* | *1.000* |
| *Venous thrombosis* | *1 (0.2%)* | *7 (0.1%)* | *0.537* |
| *Urinary tract infection* | *2 (0.4%)* | *29 (0.5%)* | *1.000* |
| *Abdominal pain* | *14 (2.5%)* | *47 (0.8%)* | *<0.001* |
| *Malnutrition* | *3 (0.5%)* | *23 (0.4%)* | *0.712* |
| *Other* | *6 (1.1%)* | *41 (0.7%)* | *0.435* |
| Serious postoperative complication^1^ | 23 (4.6%) | 168 (3.4%) | 0.152 |

*- Adjusted for matching variables

1- Serious postoperative complications were defined as a complication graded as Clavien-Dindo IIIb or higher.

| ***Table S3****. Baseline characteristics of patients with social anxiety disorder and matched controls undergoing metabolic and bariatric surgery, stratified by surgical method* | | | | |
| --- | --- | --- | --- | --- |
|  | RYGB | | *SG* | |
|  | Social anxiety group | Control group | Social anxiety group | Control group |
| *N* | *501* | *4918* | *85* | *873* |
| *Age, years* | *36.3 ± 10.2* | *36.2 ± 11.8* | *36.9 ± 10.3* | *36.4 ± 11.4* |
| *Body Mass Index, kg/m2* | *43.5 ± 5.9* | *43.4 ± 5.8* | *41.6 ± 7.5* | *41.5 ± 6.5* |
| ***Sex*** |  |  |  |  |
| *Men* | *101 (20.2%)* | *934 (19.0%)* | *13 (15.3%)* | *132 (15.1%)* |
| *Women* | *400 (79.8%)* | *3984 (81.0%)* | *72 (84.7%)* | *741 (84.9%)* |
| ***Comorbidities*** |  |  |  |  |
| *Type-2 diabetes* | *71 (14.2%)* | *653 (13.3%)* | *9 (10.6%)* | *73 (8.4%)* |
| *Hypertension* | *86 (17.2%)* | *759 (15.4%)* | *7 (8.2%)* | *106 (12.1%)* |
| *Sleep apnea* | *46 (9.2%)* | *430 (8.7%)* | *5 (5.9%)* | *51 (5.8%)* |
| *Dyslipidemia* | *46 (9.2%)* | *385 (7.8%)* | *2 (2.4%)* | *46 (5.3%)* |
| *Chronic Obstructive Pulmonary Disorder* | *19 (3.8%)* | *179 (3.6%)* | *3 (3.5%)* | *28 (3.2%)* |
| *Cardiovascular disease* | *9 (1.8%)* | *66 (1.3%)* | *1 (1.2%)* | *15 (1.7%)* |
| ***Socioeconomic factors*** |  |  |  |  |
| *Disposable income* |  |  |  |  |
| *Quartile 1* | *293 (58.5%)* | *2715 (55.2%)* | *37 (43.5%)* | *458 (52.5%)* |
| *Quartile 2* | *142 (28.3%)* | *1428 (29.0%)* | *25 (29.4%)* | *253 (29.0%)* |
| *Quartile 3* | *49 (9.8%)* | *568 (11.5%)* | *11 (12.9%)* | *104 (11.9%)* |
| *Quartile 4* | *17 (3.4%)* | *207 (4.2%)* | *12 (14.1%)* | *58 (6.6%)* |
| *Unemployment* | *58 (11.6%)* | *589 (12.0%)* | *9 (10.6%)* | *77 (8.8%)* |
| ***Origin*** |  |  |  |  |
| *Swedish born, Swedish-born parents* | *451 (90.1%)* | *4432 (90.1%)* | *73 (85.9%)* | *743 (85.1%)* |
| *2^st^ generation immigrant* | *17 (3.4%)* | *181 (3.7%)* | *6 (7.1%)* | *57 (6.5%)* |
| *1^st^ generation immigrant* | *33 (6.6%)* | *305 (6.2%)* | *6 (7.1%)* | *73 (8.4%)* |
| ***Surgical access*** |  |  |  |  |
| *Laparoscopy* | *480 (95.8%)* | *4730 (96.2%)* | *85 (100%)* | *857 (98.2%)* |
| *Conversion to open surgery* | *4 (0.8%)* | *41 (0.8%)* | *0 (0%)* | *0 (0%)* |
| *Primary open surgery* | *17 (3.4%)* | *147 (3.0%)* | *0 (0%)* | *16 (1.8%)* |

| **Table S4.** Weight loss at 1 and 2 years after Roux-en-Y gastric bypass surgery among patients with social anxiety disorder and a matched control group. | | | |
| --- | --- | --- | --- |
|  | Social anxiety group | Control group | P* |
| Weight loss at 1 year after surgery |  |  |  |
| BMI-loss, kg/m^2^ | 14.0 ± 4.27 | 14.3 ± 4.08 | 0.023 |
| Excess BMI loss (%) | 79.9 ± 25.4 | 81.9 ± 23.1 | 0.058 |
| Total weight loss (%) | 32.2 ± 8.6 | 33.0 ± 7.9 | 0.032 |
| Weight loss at 2 years after surgery |  |  |  |
| BMI-loss, kg/m^2^ | 14.7 ± 5.04 | 14.6 ± 4.75 | 0.686 |
| Excess BMI loss (%) | 83.5 ± 28.0 | 83.0 ± 24.4 | 0.866 |
| Total weight loss (%) | 33.7 ± 10.2 | 33.5 ± 9.1 | 0.793 |

*- Adjusted for matching variables

| **Table S5.** Weight loss at 1 and 2 years after sleeve gastrectomy among patients with social anxiety disorder and a matched control group. | | | |
| --- | --- | --- | --- |
|  | Social anxiety group | Control group | P* |
| Weight loss at 1 year after surgery |  |  |  |
| BMI-loss, kg/m^2^ | 11.5 ± 5.09 | 11.8 ± 4.43 | 0.192 |
| Excess BMI loss (%) | 74.4 ± 34.9 | 75.6 ± 28.4 | 0.740 |
| Total weight loss (%) | 27.3 ± 10.6 | 27.1 ± 8.9 | 0.277 |
| Weight loss at 2 years after surgery |  |  |  |
| BMI-loss, kg/m^2^ | 12.1 ± 5.50 | 11.4 ± 5.06 | 0.610 |
| Excess BMI loss (%) | 74.3 ± 29.5 | 71.6 ± 29.7 | 0.387 |
| Total weight loss (%) | 28.2 ± 10.2 | 27.1 ± 10.8 | 0.631 |

*- Adjusted for matching variables

| **Table S6.** Risk for alcohol or substance abuse, and self-harm events after surgery among patients with social anxiety disorder compared to a matched control group, stratified by surgical method. | | | | |
| --- | --- | --- | --- | --- |
|  | RYGB  HR (95% CI) | P^1^ | SG  HR (95% CI) | P^1^ |
| Alcohol or substance abuse | 2.26 (1.81-2.81) | <0.001 | 5.97 (3.03-11.78) | <0.001 |
| New onset of alcohol or substance abuse | 2.05 (1.55 – 2.70) | <0.001 | 9.85 (3.76 – 25.79) | <0.001 |
| Self-harm event | 2.41 (1.78-3.25) | <0.001 | 3.01 (1.20 – 7.55) | 0.019 |

RYGB = Roux-en-Y gastric bypass; SG = Sleeve gastrectomy; HR = Hazard ration; CI = Confidence interval

1 – Based on Cox regression adjusted for matching variables.

| **Table S7.** Health-related quality of life estimated with the Obesity related problem scale before and after Roux-en-Y gastric bypass surgery among patients with social anxiety disorder and a matched control group. | | | |
| --- | --- | --- | --- |
|  | Social anxiety group | Control group | P |
| Preoperative | 87.5 (75.0 – 95.8) | 75.0 (54.2 – 87.5) | <0.001 |
| 1 year after surgery | 33.3 (12.5 – 62.5) | 16.7 (4.2 – 37.5) | <0.001 |
| 2 years after surgery | 45.8 (16.7-70.8) | 16.7 (4.2 – 41.7) | <0.001 |

| **Table S8.** Health-related quality of life estimated with the Obesity related problem scale before and after Sleeve gastrectomy among patients with social anxiety disorder and a matched control group. | | | |
| --- | --- | --- | --- |
|  | Social anxiety group | Control group | P |
| Preoperative | 83.3 (70.8 – 95.8) | 75.0 (54.2 – 87.5) | <0.001 |
| 1 year after surgery | 33.3 (12.5 – 58.3) | 16.7 (0.0 – 37.5) | 0.003 |
| 2 years after surgery | 37.5 (6.3-64.3) | 20.8 (0.0 – 45.8) | 0.062 |
