# Supplementary material for: Metabolic and bariatric surgery among patients with social anxiety disorder, a matched cohort study
Source: PLoS One. 2026 Jan 27;21(1):e0341175. doi: 10.1371/journal.pone.0341175 (PMC12843590; doi:10.1371/journal.pone.0341175)
Supplement: Attachment — Submitted filename: rebutal_socialfobi.docx [file pone.0341175.s003.docx]

Dear Editor, Prof. Pantelis

We would like to thank the editorial board and reviewers for their comments on our manuscript. We have responded to each of the comments in the text below and made changes to the manuscript, Tables, and Supplements in response. We strongly believe that the comments from the reviewers have strengthened the manuscript. We hope that the revision matches your expectations and that it can be accepted for publication in PLoS One.

On behalf of all authors,

Erik Stenberg MD, PhD

Journal Requirements:

When submitting your revision, we need you to address these additional requirements.

1. Please ensure that your manuscript meets PLOS ONE's style requirements, including those for file naming. The PLOS ONE style templates can be found at [https://journals.plos.org/plosone/s/file?id=wjVg/PLOSOne_formatting_sample_main_body.pdf](https://eur02.safelinks.protection.outlook.com/?url=https%3A%2F%2Fjournals.plos.org%2Fplosone%2Fs%2Ffile%3Fid%3DwjVg%2FPLOSOne_formatting_sample_main_body.pdf&data=05%7C02%7Cerik.stenberg%40regionorebrolan.se%7Ce945fc6b0a77416506c708ddc820695d%7Caece5b1982274c2782181aea120ec062%7C0%7C0%7C638886763731532893%7CUnknown%7CTWFpbGZsb3d8eyJFbXB0eU1hcGkiOnRydWUsIlYiOiIwLjAuMDAwMCIsIlAiOiJXaW4zMiIsIkFOIjoiTWFpbCIsIldUIjoyfQ%3D%3D%7C0%7C%7C%7C&sdata=mfQW7k%2BqDXKyC4uww%2BID69pE%2F1vZwIDJUwBbJOb0WZ8%3D&reserved=0) and [https://journals.plos.org/plosone/s/file?id=ba62/PLOSOne_formatting_sample_title_authors_affiliations.pdf](https://eur02.safelinks.protection.outlook.com/?url=https%3A%2F%2Fjournals.plos.org%2Fplosone%2Fs%2Ffile%3Fid%3Dba62%2FPLOSOne_formatting_sample_title_authors_affiliations.pdf&data=05%7C02%7Cerik.stenberg%40regionorebrolan.se%7Ce945fc6b0a77416506c708ddc820695d%7Caece5b1982274c2782181aea120ec062%7C0%7C0%7C638886763731542558%7CUnknown%7CTWFpbGZsb3d8eyJFbXB0eU1hcGkiOnRydWUsIlYiOiIwLjAuMDAwMCIsIlAiOiJXaW4zMiIsIkFOIjoiTWFpbCIsIldUIjoyfQ%3D%3D%7C0%7C%7C%7C&sdata=xLeGlQEVD5NW79GKiUexK%2FtuHgprXhnk2SkcL%2F4XyRc%3D&reserved=0)

*Author's response: The revision has been updated accordingly*

2. PLOS requires an ORCID iD for the corresponding author in Editorial Manager on papers submitted after December 6th, 2016. Please ensure that you have an ORCID iD and that it is validated in Editorial Manager. To do this, go to ‘Update my Information’ (in the upper left-hand corner of the main menu), and click on the Fetch/Validate link next to the ORCID field. This will take you to the ORCID site and allow you to create a new iD or authenticate a pre-existing iD in Editorial Manager.

*Author’s response: Unfortunately, the ORCID ID is linked to the corresponding authors other address (*[*erik.stenberg@regionorebrolan.se*](mailto:erik.stenberg@regionorebrolan.se)*) which is probably the reason to why it could not be accepted by the system. The correct ORCID ID is* [*0000-0001-9189-0093*](javascript:popup_orcidDetail('https://orcid.org'%20,'0000-0001-9189-0093');)

3. Please include your full ethics statement in the ‘Methods’ section of your manuscript file. In your statement, please include the full name of the IRB or ethics committee who approved or waived your study, as well as whether or not you obtained informed written or verbal consent. If consent was waived for your study, please include this information in your statement as well.

*Author's response: The full name of the Swedish Ethical Review Authority is stated in the manuscript. As accepted by the Swedish Ethical Review Authority, and in line with current praxis for large, registry-based studies in Sweden, informed consent was waived for the current study which has been clearly stated in the updated version of the manuscript as requested (i.e patients are informed of the registries and that research will be conducted on the data in the registries. They can, at any time, require removal of their data from the registries (“opt-out”)).*

4. We note that you have indicated that there are restrictions to data sharing for this study. For studies involving human research participant data or other sensitive data, we encourage authors to share de-identified or anonymized data. However, when data cannot be publicly shared for ethical reasons, we allow authors to make their data sets available upon request. For information on unacceptable data access restrictions, please see [http://journals.plos.org/plosone/s/data-availability#loc-unacceptable-data-access-restrictions](https://eur02.safelinks.protection.outlook.com/?url=http%3A%2F%2Fjournals.plos.org%2Fplosone%2Fs%2Fdata-availability%23loc-unacceptable-data-access-restrictions&data=05%7C02%7Cerik.stenberg%40regionorebrolan.se%7Ce945fc6b0a77416506c708ddc820695d%7Caece5b1982274c2782181aea120ec062%7C0%7C0%7C638886763731552128%7CUnknown%7CTWFpbGZsb3d8eyJFbXB0eU1hcGkiOnRydWUsIlYiOiIwLjAuMDAwMCIsIlAiOiJXaW4zMiIsIkFOIjoiTWFpbCIsIldUIjoyfQ%3D%3D%7C0%7C%7C%7C&sdata=1NGBfYcklJ0w6AwIeHZ8rjw5RA518vSdYu2Mg8jkXlA%3D&reserved=0).

Before we proceed with your manuscript, please address the following prompts:

a) If there are ethical or legal restrictions on sharing a de-identified data set, please explain them in detail (e.g., data contain potentially identifying or sensitive patient information, data are owned by a third-party organization, etc.) and who has imposed them (e.g., a Research Ethics Committee or Institutional Review Board, etc.). Please also provide contact information for a data access committee, ethics committee, or other institutional body to which data requests may be sent.

b) If there are no restrictions, please upload the minimal anonymized data set necessary to replicate your study findings to a stable, public repository and provide us with the relevant URLs, DOIs, or accession numbers. Please see [http://www.bmj.com/content/340/bmj.c181.long](https://eur02.safelinks.protection.outlook.com/?url=http%3A%2F%2Fwww.bmj.com%2Fcontent%2F340%2Fbmj.c181.long&data=05%7C02%7Cerik.stenberg%40regionorebrolan.se%7Ce945fc6b0a77416506c708ddc820695d%7Caece5b1982274c2782181aea120ec062%7C0%7C0%7C638886763731561756%7CUnknown%7CTWFpbGZsb3d8eyJFbXB0eU1hcGkiOnRydWUsIlYiOiIwLjAuMDAwMCIsIlAiOiJXaW4zMiIsIkFOIjoiTWFpbCIsIldUIjoyfQ%3D%3D%7C0%7C%7C%7C&sdata=9Slv5m3hvzph1EIHhxv%2FX2U%2FB9eQBt6Pvn0%2BT5Gv6NY%3D&reserved=0) for guidelines on how to de-identify and prepare clinical data for publication. For a list of recommended repositories, please see [https://journals.plos.org/plosone/s/recommended-repositories](https://eur02.safelinks.protection.outlook.com/?url=https%3A%2F%2Fjournals.plos.org%2Fplosone%2Fs%2Frecommended-repositories&data=05%7C02%7Cerik.stenberg%40regionorebrolan.se%7Ce945fc6b0a77416506c708ddc820695d%7Caece5b1982274c2782181aea120ec062%7C0%7C0%7C638886763731572343%7CUnknown%7CTWFpbGZsb3d8eyJFbXB0eU1hcGkiOnRydWUsIlYiOiIwLjAuMDAwMCIsIlAiOiJXaW4zMiIsIkFOIjoiTWFpbCIsIldUIjoyfQ%3D%3D%7C0%7C%7C%7C&sdata=fjJNDowMX6CFlm5zI2r5LOtYXBCHwaSyS4I5rgNjt24%3D&reserved=0). You also have the option of uploading the data as Supporting Information files, but we would recommend depositing data directly to a data repository if possible.

Please update your Data Availability statement in the submission form accordingly.

*Author's response: The current Swedish legislation forbids sharing of pseudonymized data in public domains. However, the data is stored at Örebro University, and can be made available upon reasonable request from researchers. Previously, direct contact with the corresponding author for such requests has not been accepted by journals. Therefore, the official contacts to the registries providing the data has been listed. However, there are two reasonable options:*

1. *The corresponding author, who has direct access to the stored data, can be listed as contact person*
2. *The SOReg, which also has access to the dataset (and is an official party) can be listed as contact.*

*In the revised version, the corresponding author has been listed as contact person for such request, but this can be changed to the official SOReg contact address if the Editors prefer.*

5. You have indicated that data is available from [soreg@regionorebrolan.se, [Registerservice@socialstyrelsen.se](mailto:Registerservice@socialstyrelsen.se), [microdata@scb.se](mailto:microdata@scb.se)]. Please can we ask you to provide us with a general contact email address for the data requests, so readers can request access in perpetuity. If a general email is not available please provide a link to a website where readers can obtain access to data.

*Author's response: Please see response to Q4.*

6. We note that the grant information you provided in the ‘Funding Information’ and ‘Financial Disclosure’ sections do not match.

When you resubmit, please ensure that you provide the correct grant numbers for the awards you received for your study in the ‘Funding Information’ section.

*Author's response: These grants have been included in the disclosure to match the reports.*

7. Thank you for stating the following financial disclosure:
this work was supported by grants from Region Örebro County, Åke Wiberg Foundation, Stockholm County Council.

Please state what role the funders took in the study. If the funders had no role, please state: "The funders had no role in study design, data collection and analysis, decision to publish, or preparation of the manuscript."
If this statement is not correct you must amend it as needed.
Please include this amended Role of Funder statement in your cover letter; we will change the online submission form on your behalf.

*Author's response: Thank you! This has been included in the revised cover letter and financial disclosure to match the requirements.*

8. [Please remove all personal information, ensure that the data shared are in accordance with participant consent, and re-upload a fully anonymized data set.

Note: spreadsheet columns with personal information must be removed and not hidden as all hidden columns will appear in the published file.

Additional guidance on preparing raw data for publication can be found in our Data Policy (https://journals.plos.org/plosone/s/data-availability#loc-human-research-participant-data-and-other-sensitive-data) and in the following article: http://www.bmj.com/content/340/bmj.c181.long.](https://eur02.safelinks.protection.outlook.com/?url=http%3A%2F%2Ftrack.editorialmanager.com%2FCL0%2Fhttp%3A%252F%252Fwww.bmj.com%252Fcontent%252F340%252Fbmj.c181.long.%2F1%2F010f019806db156f-566d466c-0de1-43aa-97a3-1964236bd1f3-000000%2FJlFaARR9RLMLuKL9y_fvt_9BwxWdg4jfSUMmWHh_0Gc%3D219&data=05%7C02%7Cerik.stenberg%40regionorebrolan.se%7Ce945fc6b0a77416506c708ddc820695d%7Caece5b1982274c2782181aea120ec062%7C0%7C0%7C638886763731582212%7CUnknown%7CTWFpbGZsb3d8eyJFbXB0eU1hcGkiOnRydWUsIlYiOiIwLjAuMDAwMCIsIlAiOiJXaW4zMiIsIkFOIjoiTWFpbCIsIldUIjoyfQ%3D%3D%7C0%7C%7C%7C&sdata=65mOtM%2FrJyYmsTWrtoZkosPsBxmnk48gCwP1cgkpYOM%3D&reserved=0)

*Author's response: No personal information is included*

9. [Please include captions for your Supporting Information files at the end of your manuscript, and update any in-text citations to match accordingly. Please see our Supporting Information guidelines for more information: http://journals.plos.org/plosone/s/supporting-information.](https://eur02.safelinks.protection.outlook.com/?url=http%3A%2F%2Ftrack.editorialmanager.com%2FCL0%2Fhttp%3A%252F%252Fjournals.plos.org%252Fplosone%252Fs%252Fsupporting-information.%2F1%2F010f019806db156f-566d466c-0de1-43aa-97a3-1964236bd1f3-000000%2FBcoJ0LWW6U8Kl3ERPi6I1LloQMtuT98-AoX-VRHlOJY%3D219&data=05%7C02%7Cerik.stenberg%40regionorebrolan.se%7Ce945fc6b0a77416506c708ddc820695d%7Caece5b1982274c2782181aea120ec062%7C0%7C0%7C638886763731591564%7CUnknown%7CTWFpbGZsb3d8eyJFbXB0eU1hcGkiOnRydWUsIlYiOiIwLjAuMDAwMCIsIlAiOiJXaW4zMiIsIkFOIjoiTWFpbCIsIldUIjoyfQ%3D%3D%7C0%7C%7C%7C&sdata=9vWgD%2Fe40G3EqQT4npj6719VOScYmpQQ2khdqZA5Fbc%3D&reserved=0)

*Author's response: The information has been listed as requested.*

10. If the reviewer comments include a recommendation to cite specific previously published works, please review and evaluate these publications to determine whether they are relevant and should be cited. There is no requirement to cite these works unless the editor has indicated otherwise. 

*Author's response: Thank you for this clarification. We have acted accordingly during the revision.*

Reviewers' comments:

Reviewer's Responses to Questions

**Comments to the Author**

Reviewer #1: Major Concerns
• While the propensity matching was comprehensive for many demographic and medical factors, the SAD group differed markedly from controls in terms of baseline psychiatric profile. Notably, 69.6% of patients with SAD were on an SSRI/SNRI antidepressant (versus 27.8% of controls) and 26.8% were using benzodiazepines (versus 6.0% of controls) in the year before surgery . Prior diagnoses of substance use disorder were also more common in the SAD cohort (18.9% vs 6.5% in controls) . These differences reflect a higher burden of underlying psychiatric illness (e.g. depression, generalized anxiety) in the SAD group. Such comorbidities and treatments could themselves influence outcomes like self-harm and substance abuse relapse, independent of SAD per se. The authors have identified these baseline differences in their Results , but there is concern that they did not fully adjust for or discuss their impact. The analytic approach adjusted for the matching variables, yet psychiatric medication use and prior psychiatric history were not matching factors and thus remained imbalanced . This raises the possibility that the observed excess of self-harm and substance-related events in the SAD group might be partly driven by these pre-existing comorbid conditions rather than social anxiety alone. To strengthen their conclusions, the authors should consider additional analyses or discussion to account for these factors. For example, a sensitivity analysis controlling for baseline antidepressant use, benzodiazepine use, or prior substance abuse history would help determine if SAD remains an independent predictor of postoperative self-harm/substance outcomes after accounting for these covariates. At minimum, a more explicit discussion acknowledging that comorbid psychiatric conditions (and their treatments) could confound the relationship between SAD and adverse outcomes would be valuable.

*Author's response: This is a fair point. However, social anxiety disorder is strongly linked to many psychiatric comorbidities. Excluding these, adjusting for them, or including them as matching variables, would over adjust for mental illness, and, from our point of view, not answer the question of what outcomes can be expected for patients with social anxiety disorder. For this reason, we are also reluctant to perform further analyses stratified or adjusted for other aspects of mental health. On the other hand, we agree with the reviewer's comments that the confounding nature of other psychiatric conditions warrants further discussion and have included this in the revised manuscript. In addition, the manuscript includes stratified analyses by previous substance use. (lines 344-349)*

• The study defines SAD exposure via a recorded diagnosis in specialized healthcare, which by design captured moderate-to-severe cases and likely missed milder cases . This criterion ensures the cohort truly had clinically significant social anxiety, but it may introduce a form of selection bias. Patients with mild social anxiety (or those never diagnosed) were included among “controls,” potentially diluting group differences. More importantly, patients with the most disabling social anxiety might be underrepresented in the surgical cohort altogether – for instance, some individuals with severe SAD might avoid frequent medical appointments or opt out of surgery due to anxiety about the clinical process. The authors did not directly address how many surgery candidates with psychiatric issues drop out or are denied clearance for psychological reasons. Previous research has noted that psychological factors (including anxiety) can lead a subset of bariatric candidates to drop out before surgery . Thus, the studied population of SAD patients likely represents those who were able and willing to undergo surgery, which could mean they had adequate support or slightly less impairment in functioning than the “average” person with severe SAD. This potential selection limits generalizability – the outcomes observed may not fully apply to all individuals with SAD and obesity, especially those who never made it to surgery. The authors should discuss this context. For example, they might note that their findings apply to patients with SAD who successfully complete the surgical evaluation process, and that unmeasured factors (like level of social support or motivation) might differentiate those who underwent surgery from those who did not. A related issue is the study’s demographic specificity: the cohort was almost entirely ethnically homogenous (predominantly Caucasian in Sweden) . The authors do acknowledge this and the limited transferability to other ethnic and cultural contexts . We agree with that self-acknowledged limitation and further encourage clarifying that healthcare system differences (e.g. the structured follow-up in Sweden) might mean results could differ elsewhere.

*Author's response: We agree with the limitations in generalizability and have clarified this limitation further in the discussion. (lines 360-364)*

• The study’s mean follow-up of ~6.7 years for certain outcomes is a strength, yet complete longitudinal data on weight and HRQoL were only available up to 2 years post-surgery. The authors state that high missing data in long-term follow-up prevented evaluation of weight regain . This is a major limitation because weight regain after the first 1–2 years is common and could differ between groups. If patients with anxiety have more difficulty sustaining behavioral changes long-term, one might expect differential weight maintenance beyond the 2-year mark. In fact, external evidence suggests anxiety symptoms can predict weight outcomes over time – for example, one study found that patients with high preoperative anxiety initially lost as much weight as others at 1 year, but then regained more weight by 30 months, leading to less total weight loss at 2.5 years post-op . The present study’s finding of equivalent 2-year weight loss in SAD vs control groups is reassuring, but without data on later follow-ups we cannot know if that parity persists. The inability to assess weight regain means the long-term efficacy of MBS in SAD patients (in terms of sustained weight control) remains unaddressed. This should be highlighted as a significant limitation. The authors might consider tempering their conclusion that outcomes are “similar” in the long run, since it’s based on relatively short-term weight results. Encouragingly, they do emphasize the need for future studies on weight regain . We concur and suggest that incorporating longer-term monitoring (perhaps leveraging registry updates or linking to other data sources) would greatly enhance understanding of how anxiety disorders might impact durability of weight loss. If such data cannot be obtained for this cohort, the authors should still acknowledge that two-year outcomes do not guarantee long-term outcomes, especially in light of literature hinting at possible divergences beyond that timeframe.

*Author's response: Obtaining long-term follow-up data is certainly a key to further knowledge. However, as we have discussed in the manuscript (and as has been acknowledged by others), maintaining long-term follow-up for large populations after bariatric surgery, in particular for patients with mental health-related conditions, remains very difficult. Unfortunately, there are few other reliable sources for weight in this population. We have further emphasized the importance of long-term weight data to evaluate weight regain / recurrent weight gain for this group of patients. (lines 282 and 366-368)*

• The finding of increased “non-serious” postoperative complications in the SAD group is interesting, but some clarification is needed on this outcome. The manuscript defines a serious complication rigorously (using Clavien-Dindo ≥ IIIb: requiring reoperation, organ failure, or death) , and by inference “non-serious” complications are those below that threshold. It appears that minor complications – particularly wound issues (infections or bleeding) and abdominal pain – drove the higher complication rate in SAD patients . The authors offer a plausible hypothesis that SSRIs (taken by a large proportion of SAD patients) might contribute to bleeding risk or impaired clotting, especially if combined with anticoagulants. This mechanistic speculation is valuable. However, it would be useful to report or discuss whether differences in minor complications could also stem from behavioral factors. For instance, could patients with anxiety have a lower threshold for seeking care for issues like pain, or might they be more closely monitored, leading to higher detection of minor problems? The manuscript does not currently address this aspect. Additionally, we should ensure that the analysis of “serious” complications was not biased by missing data: since serious complications were only tracked starting in 2010 , patients who had surgery in 2007–2009 (a subset of the cohort) might not have complete data on that outcome. If those early cases were included, some serious events could be unreported, potentially underestimating serious complication rates. It’s not clear if the authors excluded 2007–2009 cases from the serious complication analysis or imputed those data. Clarifying this in the Methods would be helpful for transparency. Overall, the complication findings are noteworthy but would benefit from a bit more context. I suggest the authors explicitly note in the Discussion that no increase in life-threatening complications was observed (which is an important reassuring point), and elaborate on why minor complications were higher. Discussing both biological factors (e.g. medication effects on wound healing ) and possible psychosocial factors (e.g. health-seeking behavior) would provide a more rounded interpretation.

*Author's response: The Clavien-Dindo classification was introduced in the SOReg as a mandatory variable on January 1, 2010. As the year of surgery was included as a matching variable, it is unlikely that the exclusion of individuals operated on before 2010 would cause any bias. This has been further clarified in the revised manuscript. The potential mechanism for the increased incidence of non-serious complications has also been further discussed in response to this comment. (lines 300-302)*

Minor Concerns
• The manuscript is generally well-written, but a few sentences need minor editing for grammar/typos. For example, in the Abstract the line “patients with social anxiety disorder significantly improvement in health-related quality of life” is grammatically incorrect – it should read “experienced a significant improvement”. In the Discussion, one sentence reads “…diagnosis in specialized health care sugging a moderate to severe disease…” , where presumably “sugging” is a typo for “suggesting.” These small errors can distract readers and should be fixed. A careful proofread to catch minor spelling/grammatical issues (such as missing articles or pluralization issues) would improve overall readability. Aside from typos, most of the text is clear; just ensure consistency in verb tenses and that each abbreviation is defined at first use (HRQoL was defined properly , as was MBS, etc.).

*Author's response: Thank you for identifying these typos. The manuscript has been proofread and revised to improve readability.*

•Data Presentation – P-values and Significance: In Table 1 (baseline characteristics), some variables show very small differences between groups (e.g. a 0.2% difference in the proportion with hypertension or a 0.1 kg/m² difference in BMI) accompanied by p-values < 0.05 . The authors correctly note that there were no clinically relevant differences post-matching . It may help to explicitly indicate in a footnote that due to the large sample size, trivial differences can become statistically significant. Emphasizing clinical significance over p-value could prevent readers from overinterpreting those p-values. Similarly, when reporting outcomes, consider stating absolute rates in addition to hazard/odds ratios. For instance, the text could mention the actual incidence of self-harm in each group (e.g. “3.6 vs 1.7 events per 1000 person-years” from the results ) alongside the HR 2.44, to give the reader a concrete sense of risk difference. Minor additions like this would enhance the presentation.

*Author's response: Thank you for this comment. We agree with the reviewer that P-values should be viewed in combination with effect sizes (or similar). In Table 1 we have presented SMDs as measures of residual imbalance. All values were <0.10, suggesting a good match from the Propensity score matching. This has been explained in the methods section, but for clarity, this was also added as a footnote in the revised manuscript. The actual numbers, incidence rates, and risk measures are all presented in plain text when discussing adverse outcomes.*

• The manuscript alludes to follow-up adherence as an important factor and notes a slight trend toward lower follow-up attendance at 2 years for SAD patients (59.7% vs 63.6%, p=0.068) . It would be worth reporting the 1-year and 2-year follow-up rates more explicitly, perhaps in the Results or a table, since follow-up compliance is a key aspect of postoperative care. Currently these numbers appear in passing. Clarifying that there was no significant difference at 1 year (both ~86% follow-up) and only a non-significant drop-off difference at 2 years is useful information; it suggests that despite their social anxiety, these patients managed to engage with early follow-up nearly as well as controls. This detail supports the discussion point that careful follow-up is feasible and important in SAD patients, so highlighting it would be beneficial.

*Author's response: We agree with the reviewer that this outcome was reassuring. The results have been presented under a separate headline in the results. We would prefer not to introduce subjective comments on this outcome in the results section. Therefore, no further amendments were made to the revised manuscript.*

•Ensure consistent use of terms like “MBS” (metabolic and bariatric surgery). In some places the manuscript simply says “bariatric surgery” – which is fine – but since the title and much of the text use MBS, stick to one convention or clearly state that MBS encompasses metabolic surgery as well. This is a very minor point, but consistency avoids any confusion. Likewise, abbreviations like HRQoL, MACE (major adverse cardiovascular events), etc., are defined in the methods ; just be sure each appears with definition on first use in the main text and perhaps again in tables if they stand alone.

*Author's response: This is a good point. The manuscript has been revised for consistency.*

•A minor formatting observation – the reference list includes a StatPearls chapter (ref. 3) and some multi-line entries. Just ensure that all references follow journal style. For example, Reference 4 (Rosik, 2005) appears to be broken into two lines in the PDF ; when finalizing, check that each reference is properly concatenated. This is likely just a artifact of the PDF formatting, but worth a quick look to make sure no citations are inadvertently duplicated or truncated.

*Author's response: This appears to be an artifact from the merging. However, reference 3 was not entirely in accordance with journal style and has been updated.*

Limitations Not Acknowledged by Authors

While the authors have an appropriate limitations section discussing several key issues, there are a few additional limitations that were not explicitly addressed:
• Potential Misclassification of Controls: The study assumes that the control group has no social anxiety disorder, but because the registry only captures diagnosed cases in specialist care, some control patients might actually have undiagnosed or mild social anxiety. Such misclassification would bias the comparison toward the null (making groups more similar than assumed). The authors touched on the possibility of missed mild cases leading to underestimation of differences . They could make it more explicit that a proportion of “controls” could have subclinical anxiety that went unrecorded. This limitation is somewhat inherent to using ICD diagnoses – it might be worth acknowledging that SAD was treated as a binary, present/absent variable, whereas in reality anxiety symptoms exist on a spectrum. If some controls had social anxiety symptoms (just not formally diagnosed or treated), the true impact of having significant SAD could be larger than observed. A brief comment to this effect would be appropriate.

*Author's response: This is a good point. A short comment was added in response to this comment (lines 354-355)*

• Influence of Treatment for SAD: As noted earlier, many patients in the SAD cohort were receiving pharmacotherapy (antidepressants, anxiolytics). The therapeutic management of their anxiety could confound outcomes in different directions – for example, SSRIs might reduce some anxiety-related complications but contribute to other issues (like the wound healing discussion). The study does not separate the effects of the disorder from the effects of its treatment. One could imagine that patients with adequately treated anxiety might fare differently than those with untreated anxiety. The data to directly analyze this may not be available, but the authors should acknowledge that treatment status was not controlled. For instance, is it possible that the subset of SAD patients not on SSRIs (30% of them) had worse outcomes than those on medication? Or vice versa? This is speculative, but since virtually all SAD patients were managed for their condition in some way, the reported outcomes reflect the combination of having SAD and receiving standard psychiatric care for it. The authors might clarify that their findings apply to patients with SAD under treatment, and that outcomes could differ if a patient’s SAD were unrecognized or untreated. Recognizing this nuance is important for external validity – it suggests that ensuring patients are in treatment for their anxiety (as they were in Sweden) might be one factor in achieving good weight loss results.

*Author's response: This is a good point. The nuance has been clarified further in the limitations section (lines 360-364)*

• Lack of Direct Anxiety Outcome Measures: The study infers improvement in social anxiety symptoms from the HRQoL and the context, but it did not measure social phobia severity or anxiety levels directly after surgery. There was no specific psychiatric assessment (e.g. a social anxiety scale) administered postoperatively. This is understandable given the registry nature, but it means we don’t know how social anxiety per se changed, aside from the proxy of improved psychosocial quality-of-life. The authors themselves call for qualitative research to explore direct effects on SAD symptoms . This could be mentioned as a limitation: the study cannot confirm if bariatric surgery led to a reduction in social anxiety symptomatology, since that wasn’t directly measured. The observed HRQoL gains suggest some relief in social functioning, but targeted studies would be needed to conclude that SAD severity decreases post-surgery. By acknowledging this, the authors underline that their claim of surgery being “safe and effective” in SAD is chiefly about physical and general quality-of-life outcomes, not a cure of the anxiety disorder itself.

*Author's response: We agree with this comment and have further clarified this as an important focus for further studies (line 341-342)*

• Unmeasured Psychosocial Variables: Beyond the presence of SAD and other diagnoses, there could be other psychosocial factors not captured in the datasets that influence outcomes. For example, level of social support, coping skills, or therapist involvement post-surgery were not recorded. If SAD patients differed systematically in these (perhaps lower support networks due to their social fears, as the discussion suggests ), that could affect follow-up and mental health outcomes. The authors hint at this by discussing how social avoidance can complicate support provision . It would strengthen the limitations section to note that important factors like adherence to counseling, support group participation, or family support were not available in the registries. These could moderate outcomes (e.g. a well-supported SAD patient might not experience self-harm, whereas an isolated one might).

*Author's response: This has been addressed in response to this and an earlier comment resulting in revision of the discussion (lines 341-342, and 354-355)*

• Subgroup Analyses: The cohort included both primary surgery types (gastric bypass and sleeve gastrectomy) and both sexes, etc. The study did not report any subgroup analyses (e.g. did the effect of SAD on outcomes differ for gastric bypass vs sleeve, or for younger vs older patients?). While not strictly necessary, the absence of subgroup analysis could be noted as a limitation if interactions might be expected. For instance, alcohol misuse risk is known to be higher after gastric bypass than sleeve in general. If a large portion of the SAD group had bypass, could that partly explain the high substance use incidence? The matching was stratified by procedure, so presumably distribution was equal, and the overall HR for substance abuse was averaged across procedures. If the data allow, exploring whether one surgery type is riskier for SAD patients (in terms of mental health outcomes) could be insightful. If not, it’s a missed opportunity but not a flaw – however, mentioning that the study wasn’t powered to examine differences by surgery subtype or other subgroups would be a fair caveat.

*Author's response: While we agree with the reviewer that differences in effectiveness and safety concerning these methods are of high interest, the study was not designed to evaluate this question. In response to the comment, we have included a subgroup analysis (Tables S3-S8) and commented on this in the results section (lines 276-279) and discussion (line 284), but avoided to discuss this further as this was not intended in the original study design.*

Language and Style


There are only a few instances where language could be polished:
• As noted under minor concerns, there are a handful of typos/grammatical errors (e.g. “significantly improvement” instead of “significant improvement,” “sugging” instead of “suggesting”) . These appear to be simple mistakes that slipped through proofreading. Correcting these will enhance professionalism.

*Author's response: Thank you for identifying these errors. The manuscript has been updated to improve the quality of language throughout the manuscript.*

• A bit of attention to concise phrasing could help in a few spots. For example, the sentence in Results about SSRIs could be rephrased for clarity: “the propensity match resulted in two groups without clinically relevant differences in matched variables, but patients with SAD had higher use of SSRIs/SNRIs (69.6% vs 27.8%) and benzodiazepines (26.8% vs 6.0%) in the year before surgery” . Breaking it into two sentences or using parentheses for the percentages might improve readability. This is a minor stylistic suggestion.

*Author's response: This is a good suggestion. The text has been updated accordingly (lines 211-216).*

• The Discussion is comprehensive, but a couple of sentences are quite long. For instance, the sentence spanning lines 923–931 has multiple clauses (“Enhanced awareness and support from social networks and healthcare providers are crucial… however, the social avoidance… necessitating particular attention…”). It’s an important point; splitting it into two sentences would make it easier to follow. In general, ensure each sentence conveys one clear idea.

*Author's response: Thank you for this comment. The text has been updated for clarity (lines 318-320).*

• Consistency in tone: The tone is appropriately academic. In a few places, the wording could be slightly more precise. For example, “MBS appears to be a safe and effective treatment for severe obesity in patients with social anxiety disorders” – since “safe and effective” is a strong claim, the authors might consider adding “in the short-to-medium term” or similar, acknowledging the time frame of their data. But this is more about nuance than language correctness.

*Author's response: While we generally agree with this comment, we believe that we can support the current conclusion, which is somewhat cautious given the lack of causation that can be drawn from this non-randomized design. Therefore, we would prefer not to alter this part of the text at this stage.*


Thank you

Reviewer #2: Thank you for the opportunity to review this manuscript. The topic is highly relevant, given the worldwide prevalence of social anxiety. The study is well-structured and its objectives are clearly articulated. I have only one minor language concern: in the Abstract (lines 46-48) the main verb of the sentence is missing and should be supplied.

*Author's response: Thank you for identifying this typographic error. The text has been revised to improve the language (line 51)*

Furthermore, the paper would be strengthened by including a comparator group of patients with social anxiety who did not undergo metabolic/bariatric surgery (MBS). Such a comparison would highlight more convincingly the potential impact of MBS on this population. The marked improvement in health-related quality of life (HRQL) reported here is striking—especially in a condition where HRQL typically deteriorates over time—and deserves to be contrasted with outcomes in patients who receive no obesity treatment.

*Author's response: We fully agree with this comment. However, currently it is not possible to retrieve a fair comparator group with obesity and social anxiety disorder who did not undergo bariatric surgery.*

In my opinion the conclusion should be re-phrased in order to reflect clearly the intersting findings of this paper.
I would say: "MBS appears to be a safe and effective treatment for severe obesity in patients with social-anxiety disorder. The observed increase in minor postoperative complications—as well as the higher propensity for self-harm and alcohol or substance misuse—should not deter clinicians from recommending MBS to this population; instead, these risks highlight the importance of comprehensive pre-operative screening and structured postoperative follow-up. An individualized..."

*Author's response: Thank you for this suggestion. The conclusion has been revised to match this suggestion. (lines 373-376)*

In summary, this is a well-conceived and clinically relevant study that addresses theimportant intersection between mental health and metabolic surgery. With minor language corrections and the suggested considerations for future comparison groups, the manuscript would offer an even more compelling contribution to the literature. I congratulate the authors for their work and hope these comments will help strengthen the final version.

*Author's response: Thank you!*

Reviewer #3: Dear Authors,
I read your manuscript carefully. I found it well-written and informative. I have some comments and suggestions and I hope addressing them can increase the quality of your upcoming manuscript:

1- Please use "Metabolic and bariatric surgery (MBS)" instead of bariatric surgery in the whole manuscript including Keywords.

*Author's response: We agree with this comment and have updated the text accordingly.*

2- It can be excellent if you perform a subgroup analysis to compare SG and RYGB on anxiety disorders, HRQoL, and self-harm /suicide/ alcohol or substance abuse after MBS.

*Author's response: We agree with the reviewer on the importance of comparing SG and RYGB for patients with social anxiety disorder. In response to this comment, a supplementary subgroup analysis has been included (Table S3-8; and lines 276-279 and 284). However, as this was not part of the original study design (and not based on matched design) the results for the two methods should be viewed with caution.*

3- Some studies reported worsening of psychological disorders and substance abuse after RYGB compared to SG. Please report your results after subgroup analysis and add this point to the discussion part if this finding was found in your study or not.

*Author's response: Again, these results should be viewed with caution. However, these adverse outcomes were increased after both methods. While we would prefer not to discuss this finding in detail (as this was not part of the original study design), it has been commented on lines 312-313.*

Reviewer #4: This is a well-written manuscript presenting interesting and unique data. The findings have the potential to improve clinical care for a vulnerable patient group.

I have a few suggestions that I believe would further strengthen the manuscript:

In the paragraph on lines 80–86, please consider adding one or two additional sentences describing the characteristics of social anxiety disorder. Not all readers may be familiar with the condition, and a brief explanation—such as how social anxiety often leads to avoidance behaviors—would be appropriate.

*Author's response: An additional sentence describing the risk of isolation and social avoidance has been added in response to this comment (lines 85-86)*

The reasoning in lines 90–92 is somewhat difficult to follow. Please clarify what you mean by the connection to neuropsychiatry.

*Author's response: This mainly referred to ADHD, which has been explained in the revised manuscript. (lines 94-95)*

Regarding the Obesity-related Problems Scale: Please clarify that higher scores indicate greater problems. Most QoL scales are scored so that higher values reflect better quality of life. On line 309, you state that the group with social anxiety had lower scores, but Table 3 shows that they had higher scores, which aligns with the scale’s scoring. I suggest converting Table 3 into a figure, as this would make the results more illustrative. Also, please indicate in the figure legend (or table legend, if you choose not to convert it) that higher scores reflect poorer QoL. Be consistent in how you refer to the Obesity-related Problems Scale—sometimes it is written with a hyphen, sometimes without.

On line 308, when discussing what the Obesity-related Problems Scale measures, I suggest adding a clarification that the scale captures avoidance of social situations due to weight or body shape.

*Author's response: This is a fair point. To avoid unnecessary abbreviations, the scale was referred to by its full name. This should then be the obesity-related problems scale, which has been updated in the revised manuscript. As suggested, the data in Table 3 have been converted to a figure to better illustrate HRQoL trajectories. Additionally, the scale has been further described in the methods section (172-174) and the Figure legend to Fig 1.*

I recommend that you mention, at least briefly in the discussion, the social stigma associated with obesity and how this may interact with and potentially reinforce social anxiety.

*Author's response: Thank you for this recommendation. The stigma has been included in the revised discussion (lines 320-327)*

Individuals with a psychiatric diagnosis may be expected to have more frequent contact with healthcare services than undiagnosed individuals. Could part of the higher prevalence of self-harm and substance use diagnoses in this group be explained by more frequent follow-up and thus a greater likelihood of receiving a diagnosis?

*Author's response: The combination with psychiatric diagnoses can probably lead to either over- or under estimation, but given the high incidence of psychiatric comorbidities and established contacts with specialized psychiatric care, the major issue in this case would probably be overestimation. This has been more clearly mentioned in the revised manuscript (lines 300-302, 313-315).*

Please be more specific in your suggestions regarding how this patient group should be followed in clinical care to ensure improved management.

*Author's response: While the postoperative intervention lies beyond the scope of this manuscript, we have further discussed this on lines 320-327.*

Additional details:

Please spell out what MSD stands for in the conflict of interest section.
Some references are written in parentheses, others are not—please ensure consistency.
Line 277: “sugging” – is this a typo? Should it be “suggesting”?
Line 331: Should it be “disorders” or “disorder”?

*Author's response: The manuscript has been updated to correct these typos.*

Reviewer #5: Summary:
This manuscript reports outcomes after bariatric surgery in patients with moderate to severe social anxiety disorder (SAD), using data from Sweden’s national quality registries. The authors use propensity score matching and longitudinal follow-up to assess weight loss, complications, and psychiatric outcomes. The topic is relevant and addresses an evidence gap regarding mental health comorbidity and surgical outcomes. The methods are standard for this type of question, and the data sources are of high quality. However, several clarifications are needed before the manuscript can be considered for publication.

Major Comments:
1. Definition and Severity of Social Anxiety Disorder
The manuscript refers to patients with “moderate to severe” social anxiety disorder, but it remains somewhat unclear how this was operationalized. Please elucidate this and clarify previous reports on how registry and severity associate.
o Is it known whether the F40.1 ICD-10 diagnosis based on inpatient care, specialist outpatient care, or general practitioner records?
o Were diagnostic criteria or symptom severity scales (e.g., LSAS) available?
o Please clarify the criteria for inclusion and whether the diagnosis reflects clinically verified moderate/severe illness or simply any registered SAD code.

*Author's response: The definition was based on ICD-10 coding from the national patient registry (which is based on diagnoses of diseases and not insurance/financial claims). These diagnoses were all based on inpatient and specialist outpatient care (which has been clarified in the revised methods section, lines 125-126). While no results from scales were available for this particular study (which has been better clarified in the revised limitations section, lines 354-355), this was based on a previous validation of the same source, suggesting high validity for moderate to severe SAD.*

2. Psychiatric Comorbidities and Confounding
While the use of SSRIs and benzodiazepines is reported, other common comorbid psychiatric conditions (e.g., major depression, generalized anxiety, PTSD) are not clearly addressed.
o Please clarify whether patients with additional psychiatric diagnoses were excluded or adjusted for.
o A sensitivity analysis excluding patients with severe psychiatric comorbidities or stratifying by psychiatric medication class would strengthen causal interpretation.

*Author's response: Given the link between SAD and other psychiatric conditions, we deliberately did not match for such variables, as this would not provide a representative control group for our research question. From a clinical perspective, we also believe that this is the most interesting approach, as this answers what risks (and benefits) we can expect when counselling a patient with SAD and severe obesity. A stratified analysis, excluding patients with psychiatric disorders, could strengthen causal interpretation, but would exclude a very high number of patients, and as such, we would prefer not to include such an evaluation as this was not part of the original study design.*

Minor Comments:
1. Use of “Moderate to Severe” Terminology
If there is no standardized way the diagnosis was stratified by severity in the registry, consider avoiding this term or use wording that signifies the limitation of registry diagnosis.

*Author's response: While we fully understand this comment, the definition was based on previous validation of the data source used to define SAD in the current study. Based on this validation, it seems clear that SAD of at least moderate severity is what has been included. Thus, the study may not be representative/generalizable to patients with milder forms of SAD, which is important to recognize as previous studies have mainly evaluated outcomes among patients with milder forms of SAD. For this reason we would prefer to keep these definitions to acknowledge the generalizability of the study results. However, based on this (and previous comments) we have acknowledged the lack of information related to disease-specific scales of various forms. (lines 354-355)*

2. Discussion Section
I encourage the authors to discuss the broader clinical implications of their findings a little more, especially how the presence of SAD should prompt specific interventions in this patient population. This would help clinicians to implement changes in their own practice and significantly upgrade the clinical relevance.

*Author's response: While the effects of postoperative interventions is beyond the focus of the current study, we agree with the reviewer that such discussion may be of particular interest to the intended audience of clinicians. We have further discussed this on lines 320-327.*
