# Supplementary material for: Metabolic and bariatric surgery among patients with social anxiety disorder, a matched cohort study
Source: PLoS One. 2026 Jan 27;21(1):e0341175. doi: 10.1371/journal.pone.0341175 (PMC12843590; doi:10.1371/journal.pone.0341175)
Supplement: Attachment — Submitted filename: rebuttal_plosone.docx [file pone.0341175.s004.docx]

Dear Editor,

Thank you for considering our manuscript for publication in PLoS One. We would also like to extend our gratitude towards the reviewers for reading and commenting on our manuscript. We have addressed the additional comments from the Reviewers below and made associated revisions of the manuscript. We believe that these changes have improved the clarity and quality of the manuscript, and hope that you can consider it for publication in PLoS One.

On behalf of all authors,

Erik Stenberg

**Response to Reviewers comments:**

Reviewer #1: While the authors were generally responsive to reviewer feedback, three substantive scientific issues remain incompletely resolved:

1. The reviewer requested sensitivity analyses to determine whether social anxiety disorder remains an independent predictor of adverse outcomes after controlling for baseline psychiatric medication use (antidepressants, benzodiazepines) and prior substance abuse history.

Author Response: Declined to perform the analysis, arguing it would constitute over-adjustment for mental illness intrinsic to the SAD population. Added discussion of confounding and included stratified analysis by substance use.


The concern about whether observed adverse outcomes are attributable to SAD itself versus its associated comorbidities and treatments remains partially unresolved. The stratified analysis by substance use provides some insight but does not fully address the medication confounding issue.

*Authors response: We acknowledge this comment from the reviewer. Although we agree with the conclusion that the mechanism explaining the risks associated with SAD remains partially unresolved, this cannot fully be evaluated with the current study design, and this was also not part of the aim and design of the current study. This is a limitation that has been discussed within the manuscript and should be a focus of future studies.*

2. Weight and quality of life data only available for 2 years post-surgery, preventing assessment of long-term weight regain patterns that may differ between SAD and control groups.

Author Response: Acknowledged as a significant limitation and emphasized the need for future studies with longer follow-up. Noted the difficulty of maintaining long-term follow-up in this population.

Limits the ability to draw conclusions about long-term efficacy of bariatric surgery in the SAD population, particularly given external evidence suggesting potential divergence in weight trajectories beyond 2 years.

*Authors response: We agree with the reviewer that missing data over long-term follow-up remains a limitation. Long-term weight effects, including recurrent weight gain, is highly relevant. Unfortunately, it remains very challenging to attain long-term weight data in general after bariatric surgery, and for patients with SAD in particular. For the current study, we can only acknowledge this as a limitation and have presented this in the current manuscript.*

3.: The reviewer suggested exploring whether behavioral factors (e.g., anxiety-related differences in care-seeking behavior, lower threshold for reporting symptoms, or differential monitoring intensity) might contribute to the higher rate of non-serious complications in the SAD group.

Author Response: Discussed biological mechanisms (SSRI effects on bleeding/clotting) but provided limited discussion of behavioral factors.

Partially addressed with focus on biological rather than behavioral mechanisms.
The mechanistic understanding could be enhanced by considering both biological and behavioral pathways, but this does not fundamentally alter the study’s findings or conclusions.

*Authors response: Thank you! We agree that the likely mechanism is a combination of biological and behavioral factors. The discussion has been expanded to further discuss differences in health seeking patterns, experiences of reassurance as well as somatic symptoms related to anxiety which may be of relevance in the postoperative setting.*

Reviewer #2: Dear Authors,

In my opinion, you have addressed all the comments appropriately. I am satisfied with the manuscript in its current form, acknowledging both its strengths and limitations. I believe the insights it provides are valuable to the metabolic and bariatric surgery community. I look forward to seeing more interesting results from your database in the future.

*Authors response: Thank you!*

Reviewer #3: Dear Authors,
Thanks for addressing my comments. I think your manuscript can be publishable in PLOS One journal.

*Authors response: Thank you!*

Reviewer #4: All my comments have been addressed.
However, I noticed an inconsistent use of the abbreviation HRQoL, it is sometimes used and sometimes spelled out.
In the figure legend for Figure 1, p < 0.001 for all is stated twice. It should be sufficient to mention it once.

*Authors response: Thank you! The legends have been revised as suggested.*
